# Eddy current measurements of dielectric coating thickness on a weakly magnetic substrate within the medium frequency range

**DOI:** 10.1371/journal.pone.0340200

**Published:** 2026-01-08

**Authors:** Volodymyr Ya. Halchenko, Ruslana Trembovetska, Volodymyr Tychkov, Viacheslav Kovtun

**Affiliations:** 1 Department of Instrumentation, Mechatronics and Computerized Technologies, Cherkasy State Technological University, Cherkasy, Ukraine; 2 Department of Computer Control Systems, Faculty of Intelligent Information Technologies and Automation, Vinnytsia National Technical University, Vinnytsia, Ukraine; Manipal Academy of Higher Education, INDIA

## Abstract

A method is proposed for suppressing uncontrollable noise factors in the form of local variations in the electrophysical properties of the weakly magnetic substrate material of tested objects during eddy current measurements of the thickness of dielectric coatings in the medium frequency range up to 100 kHz. As opposed to methods focused on complicating measuring instruments, in particular the use of multi-frequency techniques, swept-frequencies, resource-intensive numerical techniques for computer-based solution of inverse problems, etc., the new approach to solving this problem involves the use of the Taguchi method for robust parameter design of optimal eddy current probes in terms of improving signal-to-noise ratios, which ultimately ensures minimal variability of the useful signal without actually eliminating uncontrollable interference inherent in the tested objects. This made the medium frequency ECPs competitive for the specified measurements compared to high frequency ones due to the simplification of the hardware part of the gauges, the removal of the problem of equipping them with special sensors, and the reduction of their cost. Because of conducting a variance ANOVA analysis, the statistical influence of the design parameters of thickness gauge probes on the characteristics of the output signal was determined. This enabled the establishment of technical requirements for the accuracy of measuring devices in the manufacturing process, as well as the maintenance of operating parameters, specifically frequency and excitation current, within acceptable limits without significantly compromising the optimal signal-to-noise ratio.

## 1. Introduction

In industry, a significant number of metal details are coated with dielectric materials. The choice of coating material is based on various considerations, in particular, corrosion resistance is provided by polymer films; weather protection – by paints and varnishes; insulation quality, its electrical strength, thermal conductivity – by glass, porcelain, rubber, epoxy resins, numerous natural and artificial polymers; decorative functions – by oxide layers. Usually, their thickness is insignificant and ranges from 5 microns to 2 mm, and only in special cases, it reaches higher values, etc. The base of the coating, i.e., the substrate, can be ferrous and non-ferrous metals, and therefore, respectively, ferromagnets and non-ferromagnets (brass, bronze, copper, aluminum alloys, etc.). With this in mind, it is advisable to distinguish parts with a dielectric coating on a weakly magnetic substrate, which do not have the pronounced properties of a ferromagnetic substrate.

The thickness of the coating is a crucial functional parameter, the control of which guarantees the appropriate quality indicators. Most often, such control is performed by non-destructive methods, in particular, magnetic ponderomotor and eddy current [1]. If the substrate is ferromagnetic, it is more efficient to use magnetic thickness gauges. Otherwise, use eddy current gauges. In thickness measurement, the most widely used are transformer surface eddy current probes (ECPs) without ferromagnetic cores, which ensure sufficiently stable temperature stability of measurements. The probe is therefore applied directly on the surface of the detail, and the measurement of the coating thickness is really reduced to determining the lift-off, since eddy currents depend on its characteristics and distance to the source of the exciting electromagnetic field. Therefore, the tested object (TO) is a planar metal surface with a thin non-conductive coating.

In general, the main factors affecting the errors in coating thickness measurements are local variations in the electrical conductivity of the substrate material, the non-uniformity of its magnetic permeability, the roughness of the surface of the substrate, and certain geometric anomalies such as surface curvature and other imperfections [2]. To accurately measure the thickness of the coating, it is very important to ensure control over the influence of these factors.

In order to overcome interference in the measurement of dielectric coatings thickness on ferromagnetic substrate, we will analyze existing approaches and techniques aimed at solving this urgent research and applied problem that is important for industry. The frequency of the electromagnetic field of excitation of the ECP determines the density of eddy currents deep into the thickness of the substrate of the TO. It is well known that weakly frequencies sensing contributes to a deeper penetration of eddy currents inside the TO, while high frequencies, on the contrary, lead to their concentration on the surface of the TO. Therefore, on the one hand, the close to perfect frequency selection automatically reduces the influence of variations in geometrical obstructive parameters, such as the thickness of the substrate material, on the measurement results, if the penetration depth of the eddy current is greater than this value. Moreover, this is quite easily achieved by using a sufficiently high frequency. On the other hand, as is known [3], the use of ECP for measuring frequencies in the range of 1–10 MHz while fixing the signal amplitude can reduce the negative impact of changes in the interfering electrophysical properties of the metal substrate of the TO. Such variations are quite significant: the variation in electrical conductivity ranges from 5 to 10% for real non-ferrous metal products, and for magnetic permeability, it can locally be 10–20%, and in some cases even 40–50%, depending on the steel grade and type of processing [2]. Thus, the suppression of interference to measurements in the form of variations in the electrophysical properties of the TO is an important problem, the solution of which significantly affects the accuracy of determining the thickness of coatings due to a significant improvement of the signal-to-noise ratio.

The transition to the megahertz measurement range, in addition to these advantages in suppressing interference, is also characterized by negative consequences [4], which makes its use not always effective and acceptable. High frequency eddy current (HFEC) measurements refer to special eddy current techniques that operate in the frequency range from 100 kHz to 100 MHz. The high frequency range of HFEC makes it suitable for measurements of special sensors and hardware. The HFEC method needs special equipment optimized for this frequency range and specific coils that are sensitive in this range. This somewhat narrows its application and makes it, to some extent, problematic, including due to the complexity of implementation and its cost.

There are other methods for overcoming this problem that are well known, which are centered on signal processing techniques of ECP and are used in the medium frequency range. This is displayed both in the processing of various informative signal parameters, in particular phase and amplitude-phase [1,5], which only partially eliminate interfering influences, and by methods of incorporating the ECP into electronic circuits. An example of this approach is the auto-generator implementation of the eddy current frequency method, i.e., the parameter method, when the ECP coil is a part of an oscillating circuit and the frequency signal due to changes serves as a carrier of useful measurement information.

Currently, researchers are also looking for effective approaches to solving this task in the direction of creating computer algorithms for numerically solving inverse problems [6,7]. The improvement of more refined techniques for obtaining more complete multiparametric information about measurement objects continues due to the use of multifrequency eddy current methods [8–11] and the use of swept-frequency [12,13]. A special category should also include pulsed eddy current techniques, the use of which in coating thickness measurement has been reported in studies [14–16]. Despite certain successes in overcoming interference factors, these methods only partially address this important problem, which still requires simpler and less expensive approaches.

A rather interesting approach, according to the authors, was proposed by them in [17], in which a study was conducted to increase values of the signal-to-noise ratio of the eddy current thickness gauge of metal plates based on a surface circular probe, where encouraging positive results were achieved using the Taguchi method. Therefore, it makes sense to scale up the research experience to the measurement of dielectric coatings on TO with a weakly magnetic substrate.

The **aim** of the study is is to create a method for suppressing noise uncontrollable factors caused by local variations in the electrophysical properties of the weakly magnetic substrate material of the TO, during eddy current measurements in the medium frequency range of the dielectric coating thickness using surface transformer probes with orthogonal rectangular coils, which leads to the increasing of the signal-to-noise ratio *S/N* as a result of Taguchi optimization of their designs.

The main contribution of this study consists, first, in formulating a new problem for eddy current thickness measurement of dielectric coatings, thanks to the optimal selection of controllable design and operating parameters of the eddy current probe, when measuring noise factors, which are local variations in the electrophysical properties of the weakly magnetic conductive base of the tested objects.

The second important contribution is a proposal to solve the formulated problem as a result of robust parameter design of probes using Taguchi method with the use of the theory of design of experiments in combination with numerical modeling to determine their output signals in order to find their optimal designs.

The third contribution is the extension of the application range of dielectric coating thickness measurements on objects with a weakly magnetic base to the medium frequency range up to 100 kHz.

Another important contribution of this study is the determination, based on ANOVA-analysis, of the hierarchy of the degree of influence of controllable parameters on the formation of thickness gauge signals, which made it possible to establish technical requirements for the accuracy of their designs and compliance with the stability of operating parameters.

The structure of the article consists of an introduction, three sections, conclusions, and a list of references.

## 2. Models and methods

### 2.1. Statement of the research

At the design stage of an eddy current gauge with a rectangular surface transformer probe, i.e., a means of measuring the thickness of a dielectric coating of a static planar TO with a weakly magnetic substrate, it is necessary to find using the Taguchi robust parameter design method, the following its operating and design parameters, which ensures stability and, accordingly, low variability of the ECP output signal and realizes the maximum signal/noise ratio without actually eliminating the effects of uncontrollable noise factors in the form of local variations in the electrophysical properties of the TO substrate materials. This is the formulation of the research problem.

**Fig 1:**
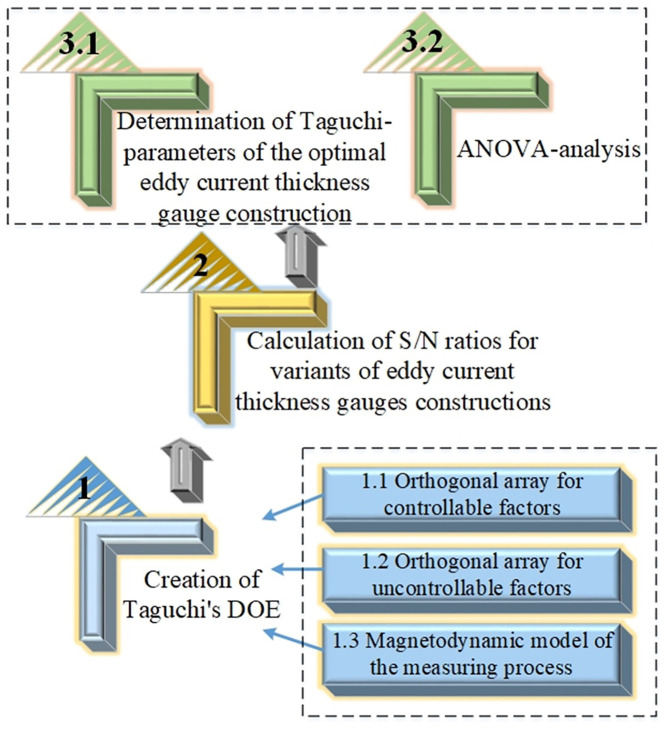
General scheme of stages for optimizing coating thickness gauges using robust parametric design.

A general scheme illustrating all the stages of the study is shown in Fig 1. It demonstrates the algorithm of robust parameter design of eddy current thickness gauges for dielectric coatings in general, the specific nature and sequence of each individual stage.

First, the Taguchi design of experiments (DOE) is created, which includes a number of components. Firstly, the controllable factors that influence the formation of the thickness gauge signal are established, and, taking into account their number from the catalog, a corresponding orthogonal array with a specified number of gradation levels is selected, and the limits of their change intervals are determined. Secondly, the same is done for uncontrollable factors. Finally, a mathematical model of the measurement process is selected. The second stage of the algorithm also involves a series of steps, namely, calculating the electromotive force (EMF) V, i.e., the output signal of the thickness gauge according to the Taguchi DOE created for each design variant for all possible combinations of noise factors. Based on the obtained V values, S/N ratios are determined, taking into account the “larger is better” property. The third stage involves searching for the optimal Taguchi parameters that provide the maximum possible S/N value and evaluating the statistical significance of the influence of the controlled parameters on the signal-to-noise ratio using Fisher’s criterion and ANOVA analysis.

In the following sub-sections, we will characterize all the main stages of the design process and quite briefly those of them that are already widely covered in specialized literature sources of information. In particular, a detailed description of the method of robust parameter design by the Taguchi method can be found in publications [18,19].

### 2.2. Magneto-dynamic model of the measurement process

**Fig 2:**
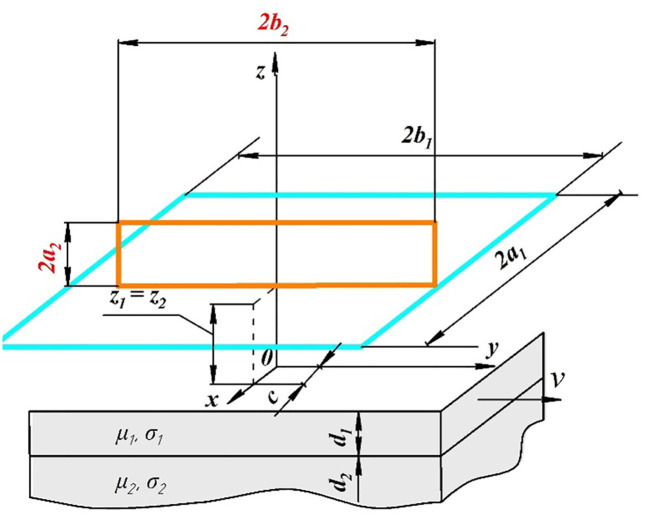
The design of the surface eddy current transformer rectangular thickness gauge of dielectric coatings.

To create a Taguchi DOE for calculating signal-to-noise ratios for variants of ECP designs, the following are necessary to calculate the output signals of the probes with variations in noise parameters. In this study, due to the ability to create a uniform distribution of eddy currents [20–23], we consider a rectangular surface transformer ECP. The design of the eddy current coating thickness gauge is shown schematically in Fig 2. The design consists of a wide rectangular excitation coil with dimensions 2*a*_1_ × 2*b*_1_ and a low-height pick-up coil of similar shape with dimensions 2*a*_2_ × 2*b*_2_. The height of both coils above the TO surface is the same and equal to *z*_1_ = *z*_2_ = 0. The current of a rectangular multi-turn coil excitation with the number of turns *N*_1_ varies according to a sinusoidal law $Ie^{j\omega t}$ with an angular frequency *ω*. The pick-up coil of the ECP has *N*_2_ turns. These coils are characterized by cross-sections *w*_1_ × *h*_1_ and *w*_2_ × *h*_2_, respectively. The excitation coil induces eddy currents in the conductor of TO, and the pick-up coil registers only the perpendicular component of the resulting electromagnetic field caused by the eddy currents.

Fig 2 is a demonstration of a more general case of measurements with a moving TO at speed *v*. However, in this article, we consider a special case of static TO, i.e., when *v* = 0. The TO consists of two layers, one with a thickness of *d*_1_ and electrical conductivity *σ*_1_ = 0 and relative magnetic permeability *µ*_1_ = 1 is dielectric, and the other is conductive weakly magnetic with a thickness of *d*_2_ and material properties *σ*_2_ and *µ*_2_. It is assumed that the TO is of infinite width and length, respectively. If the depth of penetration of the electromagnetic field is less than the thickness of the TO, then to construct a mathematical model of the ECP, we can consider a half-space, i.e., *d*_2_ = ∞. To calculate the output signal of the ECP, we chose an analytical mathematical model [24] with modifications from [25], which was used under the condition of the static state of the TO. It is created under the following assumptions: the TO environment is linear, isotropic, and homogeneous. The above studies did not take into account boundary effects, the curvature of the TO surface, the deviation of the axis of the ECP from the perpendicular to the TO surface, although their influence on the measurement of the coating thickness exists in reality.

The induced by the component of the magnetic induction of the resulting electromagnetic field *B*_*x*_, the electromotive force *V* of the surface transformer probe is calculated in accordance with the expression that defines the relationship between the controllable constructive and operating parameters of the probe, as well as uncontrollable electrophysical and geometric characteristics of the TO:


V=−jωΦ (1)

Where, $\begin{array}{cc} & \Phi =\frac{j2\mu_{0}IN_{1}N_{2}}{\pi^{2}w_{1}h_{1}w_{2}h_{2}}\int_{-\infty }^{\infty }\int_{-\infty }^{\infty }\frac{\kappa k_{1}k_{2}}{\eta^{2}\xi \varsigma^{2}}sin\left(\frac{h_{2}\xi }{2}\right)e^{-j\xi c}\times \\ & \times \left[e^{-\left(2z_{1}+w_{2}+a_{2}\right)\varsigma }-e^{-\left(2z_{1}+w_{2}+a_{2}+h_{1}\right)\varsigma }\right]d\xi d\eta , \end{array}$

$\kappa =\frac{\left(\frac{\gamma_{1}}{\varsigma }-\frac{\mu_{1}}{\mu_{0}}\right)\cdot \left(\frac{\gamma_{2}}{\gamma_{1}}+\frac{\mu_{2}}{\mu_{1}}\right)+\left(\frac{\gamma_{1}}{\varsigma }+\frac{\mu_{1}}{\mu_{0}}\right)\cdot \left(\frac{\gamma_{2}}{\gamma_{1}}-\frac{\mu_{2}}{\mu_{1}}\right)\cdot e^{-2\gamma_{1}d_{1}}}{\left(\frac{\gamma_{1}}{\varsigma }+\frac{\mu_{1}}{\mu_{0}}\right)\cdot \left(\frac{\gamma_{2}}{\gamma_{1}}+\frac{\mu_{2}}{\mu_{1}}\right)+\left(\frac{\gamma_{1}}{\varsigma }-\frac{\mu_{1}}{\mu_{0}}\right)\cdot \left(\frac{\gamma_{2}}{\gamma_{1}}-\frac{\mu_{2}}{\mu_{1}}\right)\cdot e^{-2\gamma_{1}d_{1}}}$,

$\begin{array}{c} k_{1}=\frac{\sin\left[a_{1}\xi -b_{1}\eta +\left(\xi -\eta \right)w_{1}\right]-\sin\left(a_{1}\xi -b_{1}\eta \right)}{2\left(\xi -\eta \right)}+\\ +\frac{\sin\left(a_{1}\xi +b_{1}\eta \right)-\sin\left[a_{1}\xi +b_{1}\eta +\left(\xi +\eta \right)w_{1}\right]}{2\left(\xi +\eta \right)} \end{array}$,

$\begin{array}{c} k_{2}=\frac{\varsigma \sin\eta \left(b_{2}+w_{2}\mathrm{\backslash rightleft}[e^{\varsigma \left(w_{2}+a_{2}\right)}+e^{-\varsigma \left(w_{2}+a_{2}\right)}\right]-\eta \cos\eta \left(b_{2}+w_{2}\right)\left[e^{\varsigma \left(w_{2}+a_{2}\right)}+e^{-\varsigma \left(w_{2}+a_{2}\right)}\right]}{\eta^{2}+\varsigma^{2}}+\\ +\frac{\eta \cos\left(\eta b_{2}\right)\left[e^{\varsigma a_{2}}-e^{-\varsigma a_{2}}\right]-\varsigma \sin\left(\eta b_{2}\right)\left[e^{\varsigma a_{2}}+e^{-\varsigma a_{2}}\right]}{\eta^{2}+\varsigma^{2}} \end{array}$,

$\gamma_{1}=\sqrt{\xi^{2}+\eta^{2}-j\sigma_{1}\mu_{1}\nu \eta +j\omega \sigma_{1}\mu_{1}}$, $\gamma_{2}=\sqrt{\xi^{2}+\eta^{2}-j\sigma_{2}\mu_{2}\nu \eta +j\omega \sigma_{2}\mu_{2}}$,

$\varsigma =\sqrt{\xi^{2}+\eta^{2}}$, *Ф* is the magnetic flux through the pick-up coil, *ξ* and *η* are the integration variables of the Fourier transform, $j=\sqrt{-1}$ is imaginary unit, *µ*_0_ = 4· *π*· 10^−7^ is a magnetic constant in vacuum.

The authors have developed software for calculating the EMF, which has been successfully verified by comparison with the results of finite element method calculations in the tests given in [24].

### 2.3. Selection of orthogonal arrays for controllable and uncontrollable factors

Creating a Taguchi DOE also involves the selection of certain orthogonal arrays that will determine the generated according to them, the variants of ECP designs with the corresponding operating parameters, which are generally characterized as controllable, and various combinations of uncontrollable noise parameters. A necessary condition for the adequate selection of orthogonal arrays is the previously created lists of controllable and uncontrollable parameters, which allows us to know their number in both groups. In addition, it is important to determine the number of levels of gradation for each group of these factors. All of this knowledge makes it possible to select the most suitable orthogonal arrays for specific studies from the relevant catalogs. It should also be noted that in case of exaggeration the number of factors in the selected arrays, they can be adjusted as a result of by removing unnecessary parameters without distorting their useful properties as a result of modification. Filling out the Taguchi DOE tables, in addition to choosing orthogonal arrays, involves establishing possible ranges of changes in all controllable and uncontrollable parameters, within which the search for the optimal design of ECP according to a certain criterion will be carried out. The task of the specified input data in the result determines the number of numerical experiments required to perform this search.

### 2.4. Creating a Taguchi DOE and establishing the optimal ECP construction

**Fig 3:**
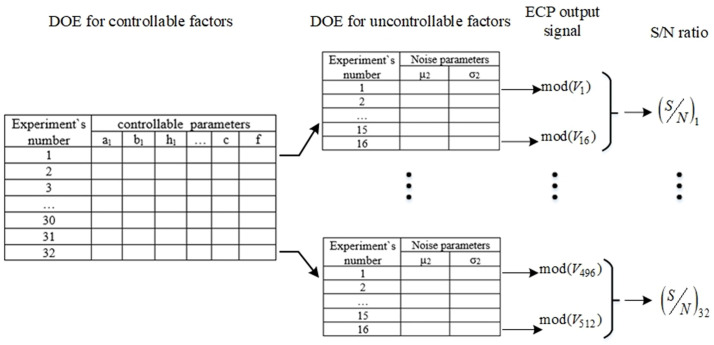
Scheme for creating a design of Taguchi’s experiments.

The Taguchi experiment design is created according to the scheme shown in Fig 3.

The calculation of the values of the output signals of the ECP is performed by formula (1). In turn, the specific signal-to-noise ratios introduced into Taguchi’s consideration, taking into account the “larger is better” property, are determined by formula (2) using the quality loss function, which is the average square deviation of the quality characteristic under study, i.e., the output signal of the ECP, from its desired target value, which is calculated based on the probe’s responses to the set of data contained in the experimental design table:


SN=-10·lg(Fitness) (2)

Where, $Fitness=\frac{\text{1}}{n}\cdot \sum_{i=1}^{n}\frac{\text{1}}{od{\left(V_{i}\right)}^{2}}$, mod(*V*_*i*_) is output signal modulus of the eddy current gauge, *n* – sample size determined by the number of interference combinations.

The evaluation of the ECP design option is carried out according to the rule: the greater the signal-to-noise ratio, the smaller the deviation of the output signal of the ECP from the target value. Based on the results obtained, conclusions are drawn regarding the best values of the controllable parameters, i.e., Taguchi parameters, which together characterize the optimal design of the probe, which provides the minimum possible variation of the output signal in the presence of interference within the specified ranges of their changes.

The final stage of the research is to conduct a variance ANOVA-analysis to determine the influence of the controllable parameters on the formation of the ECP signal, which, as a result, allows us to formulate technical requirements for the manufacture of the structure and ensure the stability of the operating parameters of the probe.

## 3. Results

Subsequently, a list of controllable and uncontrollable influencing factors was established for the eddy current thickness gauge of dielectric coatings. Thus, all geometric dimensions of the measuring part of the probe and its excitation system, as well as the center distance between them, are the design controllable parameters (Fig 2). The operating parameters include the excitation frequency and current. Then we will consider the electrical conductivity *σ*_2_ and magnetic permeability *µ*_2_ of the substrate as uncontrollable factors.

Since the study considers TO with a substrate material with weakly magnetic properties, it is advisable to first evaluate the quality of suppression of uncontrollable interference during the operation of the eddy current thickness gauge at an average frequency of about 100 kHz, and then at a high frequency of up to 1 MHz.

**Fig 4:**
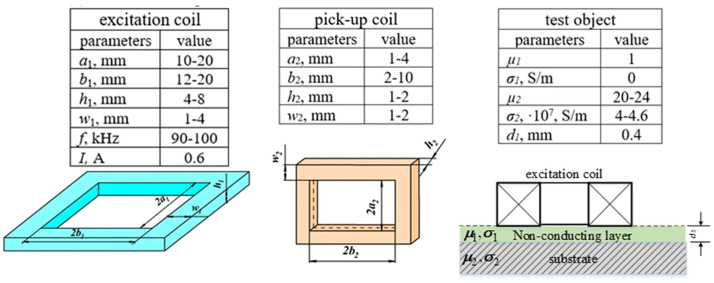
Measurement process parameters and their ranges of variation.

The next step is to determine the variation ranges of each influencing factor based on the modeling of the output signal of the ECP. For this purpose, numerical experiments were carried out to determine the dependence of the probe EMF on each of the influencing parameters. The analyzed parameter changed, while all other parameters were fixed. Then Fig 4 shows the parameters and their limits for the excitation coil, the pick-up coil, and the TO, when the gauge is operating in the medium frequency range. The number of coil turns *N*_1_ = 200, *N*_2_ = 15, and the center distance *c* = 2–9 mm. For high frequency operation of the gauge, all design parameters remain unchanged, and only the excitation frequency is set in the range from 0.9 MHz to 1 MHz.

Thus, for the two uncontrolled parameters, we chose orthogonal array L_16_(4^5^) with four levels of gradation, and array L_32_(2^1^, 4^9^) for ten controllable parameters. The following coding was used in the designation of the orthogonal array L_16_(4^5^): 4 is the number of grading levels, 5 is the possible number of influencing factors, 16 is the number of experiments; L_32_(2^1^, 4^9^) is 1 influencing factor with 2 grading levels and 9 influencing factors with 4 grading levels, 32 is the expected number of experiments on this array. For this purpose, we used their catalogs, given in [26,27]. Since the number of uncontrolled parameters in this study is less than that provided for in the L_16_(4^5^) array, it was modified to the required number of parameters; in particular, three redundant factors were removed, i.e., L_16_(4^2^) was obtained. At the same time, there is no need to modify the L_32_ array, since the number of factors provided by it corresponds to the number of factors involved in it.

The elements of the orthogonal arrays with four levels of factor gradations were recalculated into units of real physical quantities, i.e., a DOE for the interfering parameters was obtained (Table 1), which contains sixteen experiments. The DOE for the controllable parameters is shown in Table 2 with 32 experiments. At the same time, two levels of its gradation are set for the design parameter a1, and four levels for all others, which is due to the pattern of the orthogonal array L_32_(2^1^, 4^9^).

**Table 1:** The design of experiments for interfering parameters.

Experiment	1	2	3	4	5	6	7	8	9
*µ*_2_	20.00	20.00	20.00	20.00	21.33	21.33	21.33	21.33	22.66
*σ*_2_,·10^7^ S/m	4.0	4.2	4.4	4.6	4.0	4.2	4.4	4.6	4.0
**Experiment**	**10**	**11**	**12**	**13**	**14**	**15**	**16**		
*µ*_2_	22.66	22.66	22.66	24.00	22.66	22.66	22.66		
*σ*_2_,·10^7^ S/m	4.2	4.4	4.6	4.0	4.2	4.4	4.6		

**Table 2:** The design of experiments for controllable parameters.

№	*a*_1_, m	*b*_1_, m	*h*_1_, m	*w*_1_, m	*а*_2_, m	*b*_2_, m	*h*_2_, m	*w*_2_, m	*c*, m	*f*, kHz
1	0.01	0.0120	0.0040	0.001	0.001	0.0020	0.0010	0.0010	0.0020	90.00
2	0.01	0.0120	0.0053	0.002	0.002	0.0047	0.0013	0.0013	0.0043	93.30
3	0.01	0.0120	0.0067	0.003	0.003	0.0073	0.0017	0.0017	0.0067	96.60
4	0.01	0.0120	0.0080	0.004	0.004	0.0100	0.0020	0.0020	0.0090	100.0
5	0.01	0.0147	0.0040	0.001	0.002	0.0047	0.0017	0.0017	0.0090	100.0
6	0.01	0.0147	0.0053	0.002	0.001	0.0020	0.0020	0.0020	0.0067	96.60
7	0.01	0.0147	0.0067	0.003	0.004	0.0100	0.0010	0.0010	0.0043	93.30
8	0.01	0.0147	0.0080	0.004	0.003	0.0073	0.0013	0.0013	0.0020	90.00
9	0.01	0.0173	0.0040	0.002	0.003	0.0100	0.0010	0.0013	0.0067	100.0
10	0.01	0.0173	0.0053	0.001	0.004	0.0073	0.0013	0.0010	0.0090	96.60
11	0.01	0.0173	0.0067	0.004	0.001	0.0047	0.0017	0.0020	0.0020	93.30
12	0.01	0.0173	0.0080	0.003	0.002	0.0020	0.0020	0.0017	0.0043	90.00
13	0.01	0.0200	0.0040	0.002	0.004	0.0073	0.0017	0.0020	0.0043	90.00
14	0.01	0.0200	0.0053	0.001	0.003	0.0100	0.0020	0.0017	0.0020	93.30
15	0.01	0.0200	0.0067	0.004	0.002	0.0020	0.0010	0.0013	0.0090	96.60
16	0.01	0.0200	0.0080	0.003	0.001	0.0047	0.0013	0.0010	0.0067	100.0
17	0.02	0.0120	0.0040	0.004	0.001	0.0100	0.0013	0.0017	0.0043	96.60
18	0.02	0.0120	0.0053	0.003	0.002	0.0073	0.0010	0.0020	0.0020	100.0
19	0.02	0.0120	0.0067	0.002	0.003	0.0047	0.0020	0.0010	0.0090	90.00
20	0.02	0.0120	0.0080	0.001	0.004	0.0020	0.0017	0.0013	0.0067	93.30
21	0.02	0.0147	0.0040	0.004	0.002	0.0073	0.0020	0.0010	0.0067	93.30
22	0.02	0.0147	0.0053	0.003	0.001	0.0100	0.0017	0.0013	0.0090	90.00
23	0.02	0.0147	0.0067	0.002	0.004	0.0020	0.0013	0.0017	0.0020	100.0
24	0.02	0.0147	0.0080	0.001	0.003	0.0047	0.0010	0.0020	0.0043	96.60
25	0.02	0.0173	0.0040	0.003	0.003	0.0020	0.0013	0.0020	0.0090	93.30
26	0.02	0.0173	0.0053	0.004	0.004	0.0047	0.0010	0.0017	0.0067	90.00
27	0.02	0.0173	0.0067	0.001	0.001	0.0073	0.0020	0.0013	0.0043	100.0
28	0.02	0.0173	0.0080	0.002	0.002	0.0100	0.0017	0.0010	0.0020	96.60
29	0.02	0.0200	0.0040	0.003	0.004	0.0047	0.0020	0.0013	0.0020	96.60
30	0.02	0.0200	0.0053	0.004	0.003	0.0020	0.0017	0.0010	0.0043	100.0
31	0.02	0.0200	0.0067	0.001	0.002	0.0100	0.0013	0.0020	0.0067	90.00
32	0.02	0.0200	0.0080	0.002	0.001	0.0073	0.0010	0.0017	0.0090	93.30

Therefore, for each design variant, within the framework of one experiment with the specified design parameters and the specified settings for all uncontrollable factors, the probe EMF *V* is determined. To determine the optimal controllable parameters of the ECP, the signal-to-noise ratio values were used, taking into account the “larger is better” property. These calculations were performed for all experiments that are planned to be performed at the stage of creating Table 2. That is, the best controllable parameters are searched for among all levels of their gradations so that the prober signal is characterized by the lowest variability and is as insensitive as possible to uncontrollable factors.

**Fig 5:**
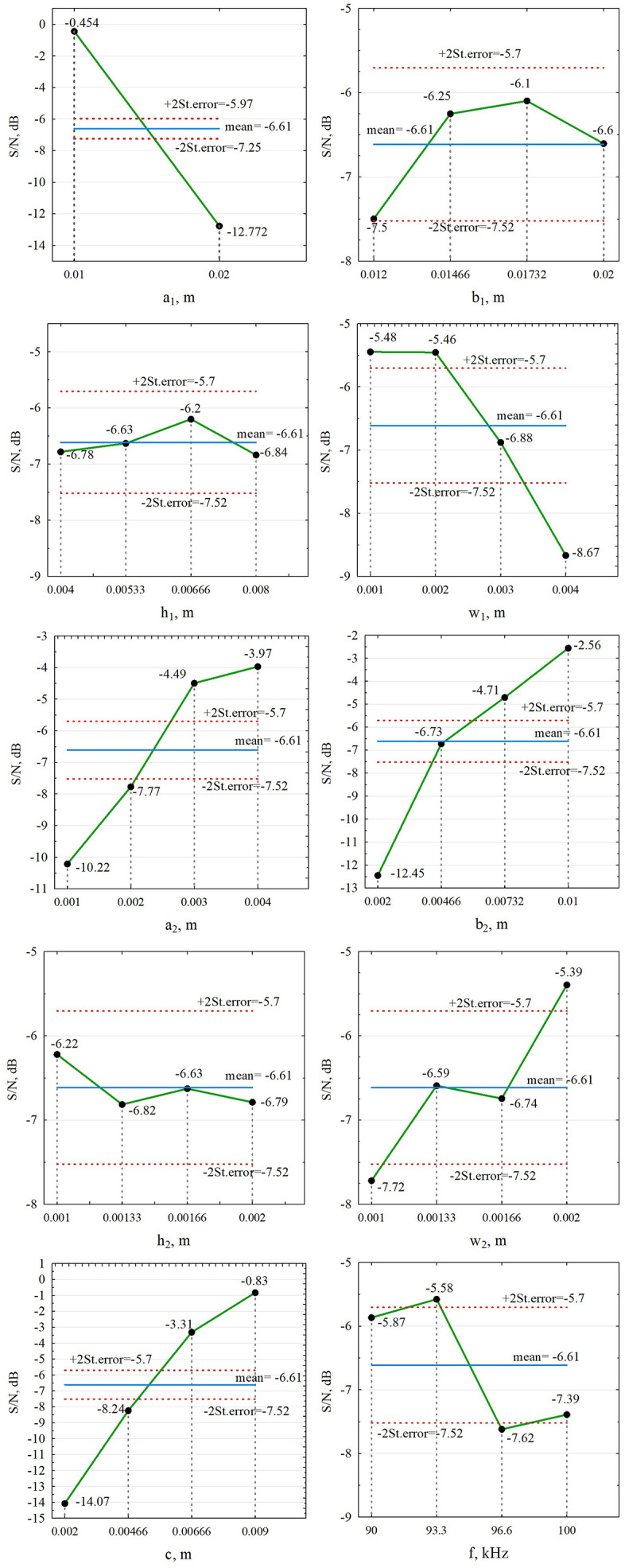
Ratio (SN‾)cont at medium frequency measurement mode.

For each controllable parameter at all levels of its gradations, the following statistical indicators are determined: their average values (SN‾)cont; absolute errors of the average value Δ=(SN‾)-(SN‾)cont, where is the average value (SN‾) of the signal-to-noise ratio of all controllable parameters; standard deviations St.Dev. For the given data (SN‾)=-6.61 dB. In the medium frequency range, the average values (SN‾)cont for each controllable parameter are shown graphically in Fig 5, with double limits of the standard error, St.error indicated.

**Fig 6:**
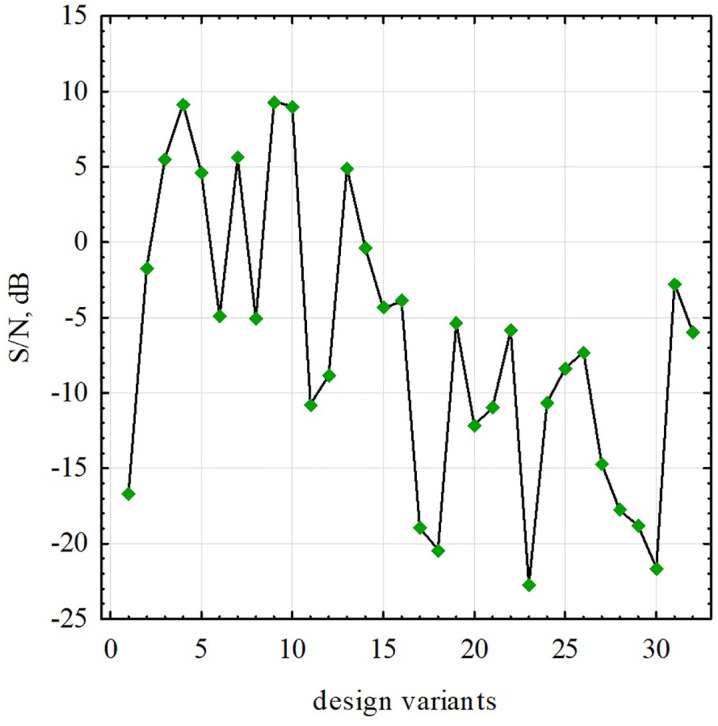
S/N values for all analyzed designs of the medium frequency eddy current gauges.

Various designs of medium frequency ECP specified in Taguchi’s design of experiments have S/N ratios values ranging from −22.7 to 9.35 dB (Fig 6).

Taking into account the previously determined statistics, the Taguchi parameters of the probe are found, i.e., its optimal values (Table 3), which provide a maximum signal-to-noise ratio of 11.26 dB in the medium frequency range. In addition, for comparison, Table 3 shows several ECP designs when measuring in the high frequency range and the *S/N* ratios that they provide.

**Table 3:** Parameters of ECP designs.

Parameter	Medium frequency ECP design	High frequency ECP designs		
		variant 1	variant 2	variant 3
*a*_1,_ mm	10.0	10.0	20.0	10.0
*b*_1,_ mm	17.3	17.3	17.3	12.0
*h*_1,_ mm	6.7	6.7	8.0	4.0
*w*_1,_ mm	1.0	4.0	2.0	1.0
*а*_2,_ mm	4.0	1.0	4.0	1.0
*b*_2,_ mm	10.0	4.7	10.0	2.0
*h*_2,_ mm	1.0	1.7	1.7	1.0
*w*_2,_ mm	2.0	2.0	1.0	1.0
*с*, mm	9.0	2.0	2.0	2.0
*f*, МHz	0.0933	0.9330	0.9660	0.9000
*S/N*, dB	11.26	10.25	1.75	4.48

**Fig 7:**
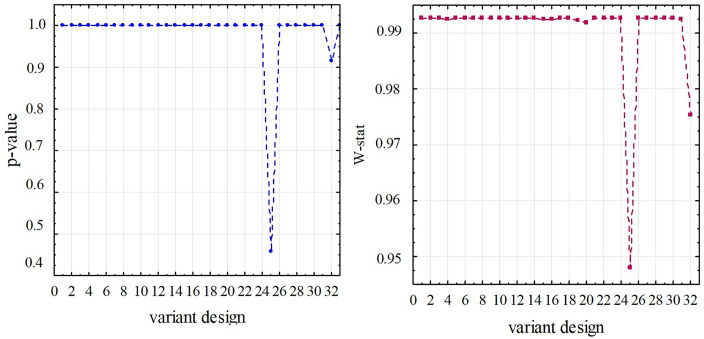
Verification of the normality of the distribution of the research data for (a) *p*-value diagram, (b) factual value of the Shapiro-Wilk criterion.

**Fig 8:**
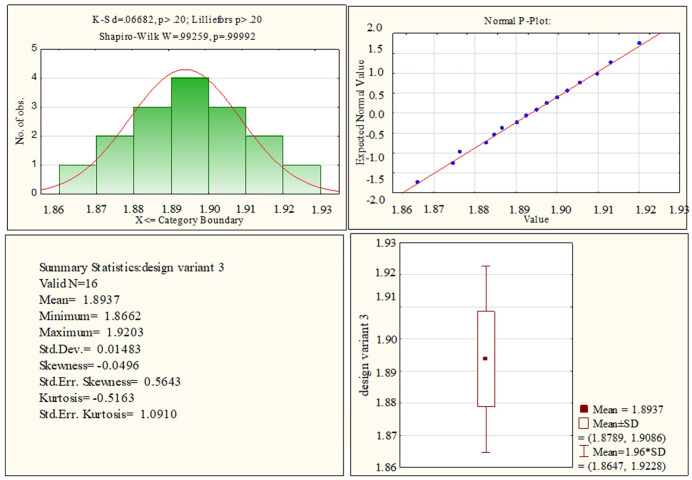
Data of normality distribution check for the design of meter No. 3 with DOE.

At the final stage of the research, based on the variance ANOVA-analysis [28,29], the statistical significance of the influence of design parameters on the SNR of the meter when it works in the middle frequency range was assessed. To this purpose, the prerequisites for the application of ANOVA have been verified in advance to ensure that the following conditions are fulfilled: the observations are independent, normally distributed, and have a homogeneous variance. That is, the initial data, namely, the values of the initial signals of the meter for each of the 32 experiments, were checked for their conformity to the normal distribution. Since each experiment has a sample of 16 elements, the most effective criterion for testing the difference between the expected theoretical and empirical distributions for small groups, i.e., the Shapiro-Wilk criterion, was used. To apply this criterion, a significance level of *α* = 5% was adopted and the value of the test statistic $W_{stat}$ was calculated, as well as the corresponding value of the probability *p* of the null hypothesis being true. Then, according to this criterion, the null hypothesis is accepted if it turns out that p>0.05, or the factual value of the criterion $W_{stat}\approx 1$. Fig 7 shows the results of testing the initial data for compliance with the normal distribution according to this criterion. Thus, according to the Shapiro-Wilk criterion, the data comply with the normal distribution in all 32 cases. In addition, Fig 8 shows graphical material in the form of a histogram of the distribution of experimental data, a normal-probability graph, and a box plot for one case out of 32 possible designs, namely for meter No. 3 with the design parameters specified in Table 2.

Then, based on the statistical indicators of sample variance *MS* and reproducibility variance *MS*_*residual*_ (Table 4), obtained by variance ANOVA-analysis and using Fisher’s criterion, the statistical significance of each factor was assessed. The influence of the assessed factor on *SNR* is insignificant if the ratio of sample variance to reproducibility variance is less than the critical value of Fisher’s criterion at the accepted significance level of *α* = 5%. In the case, where the ratio of these variances exceeds the critical value, a significant influence of the evaluated factor on the resultant feature is observed. For this research, the critical value of Fisher’s criterion is 9.27. Then, comparing the calculated values of Fisher’s criterion for each factor (Table 4) with the critical value allows us to identify statistically significant and insignificant factors and establish their hierarchy.

**Table 4:** Results of ANOVA-analysis of data for the dielectric coating thickness meter.

Parameters	*SS*	*MS*	*F*	*p*
*a*_1_	1213.9	1213.9	735.42	0.0001
*b*_1_	9.4000	3.1490	1.9074	0.3046
*h*_1_	2.0000	0.6670	0.4039	0.7620
*w*_1_	56.000	18.671	11.311	0.0383
*а*_2_	206.44	68.816	41.688	0.0060
*b*_2_	433.33	144.44	87.502	0.0020
*h*_2_	1.8000	0.6000	0.3634	0.7861
*w*_2_	21.814	7.2710	4.4048	0.1273
*c*	821.01	273.67	165.78	0.0008
*f*	25.964	8.6550	5.2428	0.1034
*residual*	4.9520	1.6510		

Where SS is the sum of squares of the variance component and residuals.

In addition, conclusions regarding the significance of parameters are also confirmed by the *p*-value. If *p* < 0.05, the hypothesis of no influence of the factor is dismissed and a conclusion is made about its statistical significance.

Four parameters, also *a*_1_, *a*_2_, *b*_2_, and *c*, were identified as having the most influence on the *SNR* of the meter on significance levels of 0.0001, 0.006, 0.002, and 0.0008.

## 4. Discusions

According to the data in Table 3, as a result of a confirmatory numerical experiment with the determined optimal parameters of the ECP design, it was established that the *S/N* level found for it exceeds all possible levels characteristic of the designs included in Taguchi design of experiments (Table 2). This is evidenced by the data in Fig 6, where the highest values of this indicator for unoptimized designs do not exceed 10 dB. Meanwhile, the optimized design provides a level of 11.26 dB.

It should also be pointed out that for high frequency ECPs, there are design options for which this indicator is even less than 5 dB. Although it should be noted that this does not guarantee that there are no cases where more efficient designs are possible. However, given the drawbacks of ECPs operating in the megahertz frequency range and considering the simplicity and advantages of measuring devices in the medium frequency range, it seems possible to expand the scope of application of simpler techniques for measuring the dielectric coating thickness with fairly high efficiency.

The variance analysis revealed the most influential design parameters of the ECP and their possible permissible inaccuracies in manufacturing. The greatest attention should be paid to maintaining the manufacturing accuracy of the excitation coil width, center-to-center distance, pick-up coil length, and its width.

Taking these into account, an assessment was made of the stability of the *S/N* ratio when exciting the ECP in the medium frequency range, provided that the other design parameters of the probe remain unchanged. The results of the study indicate a significant influence of design parameters on the *S/N* ratio, the accuracy of which varies from 10% to the optimal values. The effect on the signal-to-noise ratio of operating parameters has also been established. It should be noted that the frequency of ECP excitation has a mild effect on this indicator. However, it has been established that the specified excitation current value must be accurately maintained, as its effect is quite critical for the formation of probe signals.

## 5. Conclusions

The article formulates the problem of suppressing uncontrollable noise factors inherent in TO during measurements for eddy current thickness gauging of dielectric coatings through the optimal selection of controllable design and operating parameters of probes.

To solve this problem, we propose the use of robust parameter design of probes by the Taguchi method, using the theory of DOE to find their optimal designs in combination with numerical modeling to determine their output signals, which makes their use competitive with high frequency ECPs and accordingly expands their scope of application.

An optimal design of surface transformer probes with rectangular orthogonal coils has been obtained, which significantly reduced the variability of the output signals of the ECP without actually eliminating the interfering factors in the form of local variations in the electrophysical properties of the weakly magnetic substrate material of the TO and achieved the maximum possible signal-to-noise ratio of 11.26 dB.

In addition, the determination of the hierarchy of influence of the controllable parameters on the formation of signals of thickness gauge probes based on the variance ANOVA-analysis made it possible to establish technical requirements for the accuracy of manufacturing structures and the necessary maintenance of stable operating parameters. Thus, variations in two of the most significant design parameters, namely the width of the excitation coil and the center-to-center distance between the ECP coils, significantly affect the reduction in the signal-to-noise ratio. This is observed when the excitation coil width is increased by 10–20% from the optimal value, resulting in *S/N* changes from 6.21% to 7.5%. For the second parameter, the same changes in the direction of decrease lead to *S/N* variations from 9.94% to 15%.

Whereas failure to maintain excitation frequency stability by decreasing it by 3% from the optimum value results in a change in *S/N* of 8.7%. In addition, it is necessary to maintain a stable excitation current value, since its variation from the accepted value by 8–16% in the negative direction leads to a decrease in the signal-to-noise ratio within the range from 12.61% to 19.6%.

In the future, research is planned on the application of this approach to optimizing the design of thickness gauges for metal coatings on magnetic and non-magnetic substrates.
