# Supplementary material for: Eddy current measurements of dielectric coating thickness on a weakly magnetic substrate within the medium frequency range
Source: PLoS One. 2026 Jan 8;21(1):e0340200. doi: 10.1371/journal.pone.0340200 (PMC12782438; doi:10.1371/journal.pone.0340200)
Supplement: Attachment — Submitted filename: Rebuttal letter.docx [file pone.0340200.s002.docx]

**A REBUTTAL LETTER**

| **Reviewer #1:** | |
| --- | --- |
| We are grateful to the reviewer for their work and for pointing out the shortcomings of the manuscript of the submitted article, which allowed us to improve its content. | |
|  | |
| From what I felt able to review, regarding general methodological and linguistic areas, think the article is of interest and well written but can be much more concise. |  |
| I am not sure about the purpose of the “main attributes of the research” section. I find this is not necessary and can and should simply detail the objectives of the study. After the objectives I think that text can be removed and the information simply described in the models and methods section. You describe what you will describe in “results and discussion” but I feel it is best you just describe and there is no need to say “I will do x, y, z”. | In accordance with the reviewer's comments, section 1 has been reworked, reformatted, and corrected. |
|  |  |
| In general, I find the text to be very verbose. For example, “let us formulate the research problem as follows”, this clause is not necessary. You can simply begin with your statement, without having to say “we will make a statement…”. | The formulation of the research problem has been revised in accordance with the reviewer's comments. |
| As I mentioned originally, as I am not an expert in this topic I feel I am only able to make these general comments. But your work appears well developed and the graphics quite clear for a non-expert in the field. |  |

| **Reviewer #2:** | |
| --- | --- |
| We are grateful to the reviewer for their work and for pointing out the shortcomings of the manuscript of the submitted article, which allowed us to improve its content. | |
|  | |
| This paper focuses on the common cylindrical hole defects of locomotive traction motor shafts, concentrating on the defect detection of rotating shafts in a strong magnetic field environment, aiming to realize the identification of such defects and the diagnosis of corrosion defects. | This article is devoted to the study of **eddy current measurements of the thickness of dielectric coatings** on a weakly magnetic substrate in the medium frequency range and focuses on the suppression of all noise factors characteristic of this operation. Planar tested objects were studied. The goal was achieved using the Taguchi method. |
| After sorting out, the paper has the following issues that need to be revised and improved: |  |
| 1. The introduction section needs further refinement. It should focus on analyzing the core contributions and limitations of each scholar's research, avoiding a mere list of literature content. | Section 1, Introduction provides an overview of modern publications devoted to this problem. The main focus here is on identifying the drawbacks of previous studies. |
| 2. The expression of formulas is non-standard, and the representation method of vector symbols is incorrect. It is necessary to revise them in accordance with academic norms. | Formulas are formatted, vector quantities are not used in formulas. The corresponding mathematical relationships are presented in accordance with accepted academic norms. |
| 3. There are improprieties in the citation of references. Please check and revise them | The citation of references has been revised in accordance with the reviewer's comments. |
| 4. The corresponding relationship between charts is confusing, and some charts are missing. It is necessary to supplement and improve them and standardize their labels. | The graphic material is presented in the scope and form necessary for understanding. The symbols on the graphs are standardized in accordance with the reviewer's comments. |
| 5. The writing of the paper does not conform to academic norms. It is recommended to reorganize and revise the full text. | The structure of the submitted article is as follows: 1. Introduction, 2. Models and Methods, 3. Results. 4. Discussion, 5. Conclusions, References. |
